# Identifying a Biological Signature of Trauma-Related Neurodegeneration Following Repeated Traumatic Brain Injuries Compared with Healthy Controls

**DOI:** 10.1089/neur.2025.0052

**Published:** 2025-07-02

**Authors:** Shawn R. Eagle, Ava Puccio, Sarah Svirsky, James Mountz, Charles Laymon, Allison Borasso, Luke Henry, David O. Okonkwo

**Affiliations:** ^1^ Department of Neurological Surgery, University of Pittsburgh, Pittsburgh, Pennsylvania, USA.; ^2^ Department of Radiology, University of Pittsburgh, Pittsburgh, Pennsylvania, USA.

**Keywords:** morphometrics, neurodegeneration, plasma biomarkers, positron emission tomography, symptoms, TBI

## Abstract

The objective of this study was to compare participants at-risk for trauma-related neurodegeneration to a healthy control group on outcomes associated with Alzheimer’s disease (AD), such as subjective symptoms, neurocognitive performance, plasma biomarkers, volumetrics, amyloid-beta (Aβ) positron emission tomography (PET), and tau PET. Participants completed a comprehensive assessment protocol for neurodegenerative disease, including magnetic resonance imaging (MRI), PET scans for tau and Aβ, blood draw, subjective symptom reports related to neurodegenerative disease, and objective neurocognitive assessment. Surveys included the Neurobehavioral Symptom Inventory (NSI), Insomnia Severity Index (ISI), Epworth Sleepiness Severity (ESS), PTSD Checklist for DSM-5 (PCL-5), Brief Symptom Inventory-18 (BSI-18), Satisfaction with Life Scale (SWLS), Barratt Impulsivity Scale (BIS), and Buss Perry Aggression Questionnaire (BPAQ). PET scans were read by a neuroradiologist and rated positive or negative based upon established cutoffs. General linear models compared participants with TBI history with controls on outcomes. Age, years of education, military status, biological sex, race/ethnicity, and total self-reported TBIs were included as covariates in all models with Bonferroni corrections. Forward stepwise linear regression models were built to associate neuroimaging outcomes with symptom domains; inclusion in the linear regression required a *p* value <0.1. The average age for both groups was ∼40 years. The TBI group reported an average of five TBIs; the control group reported an average of one TBI. Across seven regions of interest, only one TBI participant met established PET cutoffs for neuropathology in one cortical region. After controlling for age, sex, race/ethnicity, years of education, military status, and TBI history, there were no statistically significant differences between groups in any neurocognitive outcome (*p* = 0.06–0.95), Aβ or tau PET (*p* = 0.05–0.70), MRI volumetrics (*p* = 0.06–0.98), or plasma biomarkers (*p* = 0.06–0.85). The TBI group had higher NSI, PCL-5, BSI-18, BPAQ, ESS, and ISI scores compared with the controls (*p* < 0.001–0.042). Within the TBI group, amygdala normative percentile and/or amygdala asymmetry index were included in the final models for NSI, SWLS, PCL5, BIS, BPAQ, and ISI. Only two models included a statistically significant PET outcome in the final model. In this sample with a mean age of 40 and a history of 5+ TBIs, core diagnostic biomarkers for AD were not different from controls despite significantly higher symptom burden. Volumetrics in critical brain regions were associated with several symptom domains in the TBI group, indicating that cortical volumetrics (especially in the amygdala) may be a more viable early biomarker of chronic symptom burden in this population than PET scans.

## Introduction

The Defense and Veterans Brain Injury Center reports military personnel have suffered >468,000 traumatic brain injuries (TBIs) since 2000.^1^ A primary concern for those who repeatedly suffer TBI and/or repeated head impacts is increased risk for later life neurodegenerative disease.^2^ Identifying biomarkers which signal potential neurodegenerative changes to the brain may provide an opportunity to initiate treatments and mitigate further neurodegeneration. However, while neurodegenerative disease risk is increased following TBI, there are no established criteria for identifying *in vivo* neurodegeneration secondary to head trauma. As a result, patients with increased risk for trauma-related neurodegeneration are often compared with standardized binary (yes/no) criteria for other diseases, such as Alzheimer’s disease (AD), which may increase risk for false negative results in this population.^3^

Research using positron emission tomography (PET) scans demonstrates that deposition of Aβ plaques (a key *in vivo* hallmark for AD) occur in a spatial-temporal pattern in both cognitively impaired and cognitively unimpaired adults.^3,4^ Aβ PET is considered the earliest detectable biomarker of AD.^5^ Importantly, early stages of Aβ accumulation occur with little-to-no clinical symptoms or impairments.^6^ A positive PET scan for AD using the ligand Pittsburgh Compound B (PiB) must have moderate to frequent Aβ plaque density in at least one of four regions: frontal lobe, posterior cingulate, precuneus, or parietal lobe.^7^ Volume asymmetry between critical brain regions (e.g., limbic system) is another early indicator of AD and can distinguish between AD and healthy aging.^8–10^ Understanding the temporal stages of the AD continuum has become a primary focus of research as pre-clinical detection of sequential Aβ plaque deposition may provide a therapeutic window to reduce disease progression.^11,12^ These principles theoretically apply to those at risk for trauma-related neurodegeneration, but these data are not currently available. The purpose of this study was to compare participants at-risk for trauma-related neurodegeneration with a healthy control group on outcomes associated with neurodegenerative disease, such as subjective symptoms, neurocognitive performance, plasma biomarkers, magnetic resonance imaging (MRI) volumetrics, Aβ PET, and tau PET. Neuroimaging biomarkers (i.e., MRI volumetrics, Aβ PET, and tau PET) were correlated with clinical measures to understand the association between underlying biological processes after a history of repeated TBIs and clinical presentation.

## Methods and Materials

### Design and participants

Participants completed a 1–2-day comprehensive assessment protocol for neurodegenerative disease, including MRI, PET scans for tau and Aβ, blood draw, subjective symptom reports related to neurodegenerative disease, and objective neurocognitive assessment. Inclusion criteria for the TBI group were: 1. History of 1+ TBIs that occurred ≥1 year prior to enrollment; 2. Age 29–59 years; 3. Suspected sign as cognitive impairment, operationally defined as one or more of the following: Neurobehavioral Symptom Inventory (NSI) score ≥6 on the 4 cognitive NSI items and/or AD8 score ≥2, performance on a neuropsychological assessment in the borderline/impaired range, professional diagnosis of cognitive impairment. Exclusion criteria for the TBI group were: 1. History of penetrating TBI; 2. History of pre-existing neurological or neurodegenerative disorder; 3. History of psychiatric disorder; 4. Active drug or alcohol dependence; 5. Contraindication to MRI or PET scans. Inclusion criteria for the control group were age 29–59 years and AD8 score ≤1, and exclusion criteria were a history of blast exposure or moderate to severe TBI. This study was reviewed and approved by the University’s Institutional Review Board for human subject research. This article was structured within the Strengthening the Reporting of Observational Studies in Epidemiology (STROBE) guidelines.

### Subjective symptom reports

The NSI is a 22-item self-reported measure of neurobehavioral symptoms.^13^ Total NSI score was analyzed with higher scores indicating worse post-concussive symptoms. The Insomnia Severity Index (ISI) is a 7-item questionnaire where participants rate the nature and symptoms of insomnia.^14^ The Epworth Sleepiness Severity (ESS) survey is a questionnaire about symptoms and impairments related to daytime sleepiness.^15^ The PTSD Checklist for DSM-5 (PCL-5) is a 20-item measure assessing 20 symptoms of post-traumatic stress from the DSM-5.^16^ Total PCL-5 score was analyzed with higher scores indicating more post-traumatic stress symptoms (range: 0–80). The Brief Symptom Inventory-18 (BSI-18) was used to assess somatization, depression, and anxiety symptoms.^17^ Each domain has six individual items that are summed for a total subscale score within that domain and then converted into T scores. The total BSI-18 T score was analyzed, with higher scores indicating more psychological health symptoms (range: 0–100). The Satisfaction with Life Scale (SWLS) is a short self-reported questionnaire of how satisfied someone is with their life.^18^ Total SWLS score was analyzed with higher scores indicating greater life satisfaction (range: 5–35). The Barratt Impulsiveness Scale (BIS) is an 11-item measure of self-reported impulsive behaviors.^19^ Total BIS score was analyzed with higher scores indicating greater levels of impulsivity. The Buss Perry Aggression Questionnaire (BPAQ) is a 20-item self-reported scale of aggressive behavior.^20^ Total BPAQ score was analyzed with higher scores indicating greater levels of aggression.

### Objective neurocognitive assessments

The Controlled Oral Word Association Test is an assessment of verbal fluency, where participants are given three letters and are asked to name as many words as possible beginning with one of those letters in a 1-min period.^21^ Participants are also asked to name as many animals as possible in a 1-min period. The total number for each subtest was analyzed as a normative percentile.

The California Verbal Learning Test is a neurocognitive test that measures a participant’s ability to learn and remember verbal information.^22^ An examiner reads aloud a list of 16 nouns at slightly longer than 1-sec intervals in fixed order over five learning trials. Immediately after each trial, the participant is asked to recall as many words as possible. Free and cued recall of the first word list is conducted immediately (short delay) and again after 20 minutes (long delay). A recognition task is conducted at the end in which the examiner reads aloud a 44-word list and the participant indicates whether it is one of the previously used words or a distractor word.^23^

The Wechsler Adult Intelligent Scale 4^th^ edition is an assessment of cognitive ability.^24^ The processing speed index, which reflects a participant’s visual motor speed during a symbol search and coding subtest, was analyzed as a normative percentile.

The Trail Making Tests A and B (TMT-A and TMT-B) measure psychomotor speed, attention, and executive function.^25^ TMT-A requires attention and visual search to draw a line connecting randomly placed numbers in order from 1 to 25 as quickly as possible. TMT-B requires executive function to set-shift by drawing a line connecting numbers and letters in an alternating fashion (1-A-2-B and so on) until they reach the number 13. Scores are based on time to complete the tasks.

The Rey Complex Figure Test measures visuo-constructional ability and visual memory.^26^ The Rey copy presents an abstract picture to the participant which they must draw on a separate blank sheet of paper, in as close to the original design as possible. Following the copy trial, an immediate recall trial is given to have the participant draw the figure from memory. Twenty minutes later, they are again asked to draw the figure from memory for the delayed recall.

The Stroop test is a neuropsychological assessment to evaluate a participant’s ability to inhibit cognitive interference that occurs when processing two stimuli at the same time.^27^ Specifically, participants are presented with color words printed in different ink colors and asked to name the color of the ink. The interference effect was converted to a T score and analyzed.

### MRI morphometry

A standard 3T MRI scan was performed on consenting participants. The scan was analyzed with NeuroQuant software that identifies and segments brain structures.^28^ NeuroQuant compares the calculated volumes of each brain structure to a database of normative values based upon the participant’s age, gender, and cranial volume. Due to their established relationship to neurodegenerative diseases, normative percentiles for the amygdala, hippocampus, parahippocampus, thalamus, posterior cingulate, entorhinal cortex, and temporal pole were analyzed. Asymmetry indices for the amygdala, hippocampus, and thalamus were also analyzed, where a positive value indicates a larger right-sided structure and a negative value indicates a larger left-sided structure.

### PET imaging

The ligand [^18^F]AV-1451 was prepared according to methods described by the manufacturer. [^11^C]PiB was synthesized by published methods developed by our group.^29,30^ PET image data were acquired on a Siemens Biograph mCT Flow™ TrueV PET/CT scanner (Siemens Medical Solutions USA, Malvern, PA). For this study, all data were reconstructed using filtered backprojection with time-of-flight correction on a 256 × 256 voxel grid with voxel dimensions of 1.27 × 1.27 × 3.0 mm. Fifteen mCi of high specific activity (>0.5 Ci/μmol at time of injection) [^11^C]PiB were injected over 20 sec. The participant relaxed for 30 min, after which they are positioned in the scanner and the head immobilized using a thermoplastic mask. A low-dose computed tomography (CT) was acquired for attenuation correction, followed by a 30 min PiB PET scan (6 × 5-min frames) acquired over the 40–70 min post-injection interval. Following the completion of the [^11^C]PiB scan, subjects were removed from the scanner and allowed a 15-min break, after which time 10 mCi of [^18^F]AV-1451 was administered intravenously. Approximately 65 min after [^18^F]AV-1451 injection, the subject was positioned in the Siemens mCT scanner (quiet dimly-lit room) with imaging planes parallel to the cantho-meatal line and primary areas-of-interest (including cerebellum) within the central 7 cm of the field of view, for which the 3D sensitivity of the tomograph is most uniform.^31^ A second low-dose (16 mrem) CT scan (19 mAs, 120 kVp, 21.6 cm axial scan length) was obtained for CT based-attenuation correction of emission data. Following the CT scan, [^18^F]AV-1451 PET emission data were collected in six 5-min frames covering the 75 to 105 min post-injection interval.

PMOD software was used for motion correction, PET-to-MR registration, region-of-interest (ROI) sampling and partial volume correction. Correction for subject motion during the multi-frame PET scan was performed using a frame-to-frame registration procedure. The anatomical MPRAGE MRI was reoriented along the anterior-posterior commissure and the averaged PET images was co-registered to the reoriented MRI. Freesurfer software was applied to perform magnetic resonance bias correction, automated ROI parcellation and tissue segmentation. A Freesurfer parcellation template was applied in PMOD to a sample five primary cortical ROIs that are most relevant to Aβ pathology (i.e., anterior cingulate gyrus, frontal cortex, lateral temporal cortex, parietal cortex, and precuneus). ROI sampling also includes the anterior medial temporal lobe that is most relevant to tau pathology (composed of entorhinal cortex, amygdala, anterior hippocampus and parahippocampal gyrus [excluding choroid plexus]), somatosensory cortex (SMC) as a control ROI for negligible [F-18]AV-1451 uptake, and cerebellum (CER) as general nonspecific reference region. Regional standardized uptake value (SUV) measures are computed for all radiotracers by normalizing tissue uptake to injected radioligand dose and body mass (unitless, assuming 1 g/cm^3^ tissue density). Each regional SUV (or SUV image) is normalized to the CER reference SUV to generate SUV ratio (SUVR or SUVR image) outcomes. The SUVR is a simple tissue-ratio approach for determining brain Aβ load that compares favorably to fully quantitative analyses.^32^ Partial volume correction of the PET data (regional and voxel) was performed using the Rousset method.^33,34^

Amyloid status (Aβ(+/−)) is assessed using a [^11^C]PiB PET average cortical global cutoff and individual regional cutoffs that were determined using statistical clustering, compared with visual reads and supported correlational analyses between PIB PET and neuropathology measures of Aβ.^35^ The same systematic approach is applied to determine tau(+/−) cutoffs for [^18^F]AV-1451, such as an ROI SUVR value more than 2 standard deviations above the mean SMC SUVR that serves as a control ROI for negligible [^18^F]AV-1451 uptake. PET scans were interpreted qualitatively by a clinical neuroradiologist investigator as “definitely normal,” “probably normal,” “probably abnormal,” or “definitely abnormal.”

### Blood biomarker processing and detection

Sample collection took place during the assessment period and plasma aliquots were stored at −80°C until analysis. Thirty plasma samples (n = 8 controls, n = 22 TBI) were run on the Alamar NULISA™ CNS Diseases panel (Alamar Biosciences, Fremont, CA), as previously described.^36,37^ Briefly, the immunoassay uses a pair of capture and detection antibodies of each target, proximity ligation to generate a DNA reporter molecule that is quantified using next-generation sequencing. This allows for high analyte specificity and sensitivity of multiplexed detection of neurodegenerative-associated markers. Amyloid-beta (Aβ) 38, Aβ 40, Aβ 42, phosphorylated (p) Tau-181, pTau-217, pTau-231, and phosphorylated transactive response DNA-binding protein 43 (pTDP-43) were measured using the automated Alamar Argo HT workflow. Sample controls, internal controls, inter-plate controls, and negative controls were all included to assess assay performance and meet quality control standards. Analyte concentration is log2 transformed and values are defined as NULISA Protein Quantification units based on internal controls.

### Statistical analysis

General linear models were built to compare participants with TBI history with controls on symptoms, neurocognitive performance, Aβ/tau PET uptake, morphometrics, and plasma proteins related to neurodegenerative disease. Age, years of education, military status (yes/no), biological sex (male/female), race/ethnicity (white, Black or Asian), and total self-reported TBIs were included as covariates in all models. Bonferroni corrections were applied for multiple comparisons. Partial eta-squared (ηρ^2^) effect sizes are reported for all comparisons. Due to small sample sizes, each model underwent a bootstrap procedure with 1,000 iteration resampling. Estimated marginal means and standard errors are reported for each variable. Forward stepwise linear regression models were built to associate neuroimaging outcomes with symptom domains (e.g., NSI, SWLS, PCL5, BSI-18, BIS, BPAQ, ESS, and ISI). Inclusion in the linear regression required a *p* value <0.1. Standardized coefficients (β) are reported for variables included in the final models. Adjusted *R*^2^ is reported for each model. Alpha was set to *p* < 0.05. Variance inflation factors (VIFs) for all included predictors were evaluated post-hoc for multicollinearity. All VIFs were <4, indicating multicollinearity was not a concern.

## Results

Descriptive statistics for the overall sample can be viewed in Table 1. The average age for both groups was 40 years, with the TBI group being 82.6% male and the control group being 90% male. Both groups were predominantly white race/ethnicity. The TBI group was 60.8% Veterans while the control group was 10% Veterans. The TBI group was 29.3 ± 10.2 years from the first TBI and 10.5 ± 6.8 from their last TBI. Neither Aβ nor tau PET scans for either group met established cutoffs for neuropathology.

**Table 1.** Descriptive Statistics for the Overall Sample

	TBI (*n* = 23)	Controls (*n* = 10)
Age	40.3 ± 8.4	39.7 ± 7.9
Biological sex		
Male	19 (82.6%)	9 (90.0%)
Female	4 (17.4%)	1 (10.0%)
Race/ethnicity		
White	22 (95.7%)	9 (90.0%)
Black	0 (0.0%)	1 (10.0%)
Asian	1 (4.3%)	0 (0.0%)
Years of education	15.2 ± 1.9	16.6 ± 2.3
Military status		
Veteran	14 (60.9%)	1 (10.0%)
Civilian	9 (39.1%)	9 (90.0%)
Prior TBIs	5.8 ± 3.3	1.0 ± 1.1
Years since first TBI	29.3 ± 10.2	29.8 ± 6.9
Years since last TBI	10.5 ± 6.8	27.8 ± 6.5

Between-group comparisons for self-reported symptoms can be viewed in Table 2. After controlling for age, sex, race/ethnicity, years of education, military status, and TBI history, the TBI group had higher NSI, PCL-5, BSI-18, BPAQ, ESS, and ISI scores compared with controls (*p* < 0.001–0.042). There was no difference between groups in SWLS or BIS (*p* = 0.063–0.431). Qualitative interpretation of PET scans can be viewed in Supplementary Table S1. All regions of interest for the TBI group were rated as “definitely normal” for all participants, with the exception of four participants rated as “probably normal” for tau in the temporal lobe, one rated as “probably normal” in the frontal lobe, and one rated as “probably abnormal” in the frontal lobe.

**Table 2.** General Linear Model Comparing Participants with Repeated Traumatic Brain Injury History and Controls on Subjective Symptom Surveys

	TBI (*n* = 23)	Control (*n* = 9)	ηρ^2^	*p*
NSI	31.5 ± 2.4	11.1 ± 4.4	0.40	0.002*
SWLS	19.0 ± 2.2	28.9 ± 3.9	0.17	0.063
PCL5	35.4 ± 2.3	5.0 ± 4.1	0.64	<0.001*
BSI	63.0 ± 1.5	45.2 ± 2.7	0.58	<0.001*
BIS	65.4 ± 3.0	59.8 ± 5.4	0.03	0.431
BPAQ	75.8 ± 4.0	55.5 ± 7.2	0.20	0.042*
ESS	9.4 ± 0.9	4.3 ± 1.6	0.25	0.021*
ISI	25.0 ± 2.0	12.8 ± 3.6	0.27	0.016*

NSI, Neurobehavioral Symptom Inventory; ISI, Insomnia Severity Index; ESS, Epworth Sleepiness Severity; PCL5, PTSD Checklist for DSM-5; BSI, Brief Symptom Inventory; SWLS, Satisfaction with Life Scale; BIS, Barratt Impulsivity Scale; BPAQ, Buss Perry Aggression Questionnaire. ^*^statistically significant at *p* < 0.05.

Between-group comparisons for neurocognitive assessments can be viewed in Table 3. After controlling for age, sex, race/ethnicity, years of education, military status, and TBI history, there were no statistically significant differences between groups in any neurocognitive outcome (*p* = 0.06–0.95).

**Table 3.** General Linear Model Comparing Participants with Repeated Traumatic Brain Injury History and Controls on Objective Neurocognitive Assessments

	TBI (*n* = 23)	Control (*n* = 9)	ηρ^2^	*p*
COWAT letters percentile	38.2 ± 6.2	57.9 ± 11.6	0.07	0.20
COWAT animals percentile	38.5 ± 5.5	48.6 ± 10.2	0.02	0.45
CVLT short delay free	49.5 ± 2.7	53.8 ± 5.0	0.02	0.50
CVLT short delay cued	53.7 ± 2.7	53.2 ± 5.0	0.00	0.93
CVLT long delay free	50.1 ± 2.7	53.9 ± 5.1	0.01	0.56
CVLT long delay cued	50.0 ± 2.6	53.9 ± 4.8	0.02	0.53
CVLT recognition discrimination	50.0 ± 2.5	47.9 ± 4.6	0.01	0.73
WAIS PSI percentile	45.1 ± 5.6	71.4 ± 10.5	0.14	0.06
TMT-A	53.8 ± 2.6	51.2 ± 4.8	0.01	0.68
TMT-B	49.2 ± 2.2	55.4 ± 4.2	0.05	0.26
Rey immediate	52.1 ± 2.8	48.5 ± 5.3	0.01	0.59
Rey delay	51.5 ± 3.1	44.6 ± 5.8	0.04	0.35
Stroop interference	51.3 ± 2.1	51.6 ± 3.8	0.00	0.95

CVLT, California Verbal Learning Test; COWAT, Controlled Oral Word Association Test; PSI, processing speed index; WAIS, Wechsler Adult Intelligent Scale; TMT, Trail Making Test.

Between-group comparisons for PET tau and Aβ uptake can be viewed in Table 4. After controlling for age, sex, race/ethnicity, years of education, military status, and TBI history, there were no statistically significant differences in Aβ or tau SUVRs between groups (*p* = 0.05–0.70).

**Table 4.** General Linear Model Comparing Participants with Repeated Traumatic Brain Injury History and Controls on Positron Emission Tomography Standardized Uptake Value Ratios for Amyloid Beta and Tau

	TBI (*n* = 17)	Control (*n* = 5)	ηρ^2^	*p*
Aβ superior frontal	1.03 ± 0.01	1.08 ± 0.02	0.25	0.05
Aβ orbitofrontal	1.14 ± 0.02	1.09 ± 0.05	0.05	0.42
Aβ lateral temporal	1.03 ± 0.01	1.06 ± 0.02	0.07	0.33
Aβ parietal	1.06 ± 0.01	1.05 ± 0.03	0.01	0.70
Aβ posterior cingulate	1.13 ± 0.02	1.19 ± 0.04	0.10	0.24
Aβ precuneus	1.11 ± 0.01	1.14 ± 0.03	0.03	0.52
Tau Braak 1–2	1.09 ± 0.03	0.95 ± 0.06	0.19	0.09
Tau Braak 3–4	1.07 ± 0.02	0.99 ± 0.04	0.18	0.10
Tau Braak 5–6	1.00 ± 0.01	0.96 ± 0.03	0.08	0.30

Between-group comparisons for MRI morphometry can be viewed in Table 5. After controlling for age, sex, race/ethnicity, years of education, military status, and TBI history, there were no statistically significant differences between groups in MRI morphometrics (*p* = 0.06–0.98).

**Table 5.** General Linear Model Comparing Participants with Repeated Traumatic Brain Injury History and Controls on Magnetic Resonance Imaging Volumetrics

	TBI (*n* = 17)	Control (*n* = 5)	ηρ^2^	*p*
Hippocampus asymmetry index	−3.1 ± 1.1	−2.2 ± 2.0	0.01	0.72
Amygdala asymmetry index	12.4 ± 1.9	4.2 ± 3.6	0.12	0.09
Thalamus asymmetry index	−4.0 ± 1.0	−0.03 ± 1.8	0.10	0.12
Hippocampus percentile	62.6 ± 5.7	74.4 ± 10.6	0.03	0.39
Entorhinal cortex percentile	35.1 ± 7.0	41.2 ± 13.1	0.01	0.72
Thalamus percentile	86.5 ± 5.0	91.7 ± 9.4	0.01	0.67
Posterior cingulate percentile	74.6 ± 5.7	47.7 ± 10.6	0.14	0.06
Parahippocampus percentile	46.0 ± 5.8	28.2 ± 10.7	0.07	0.21
Temporal pole percentile	38.6 ± 58.6	58.6 ± 13.6	0.05	0.26
Amygdala percentile	45.8 ± 5.6	45.4 ± 10.4	0.00	0.98

Between-group comparisons for plasma biomarkers can be viewed in Table 6. After controlling for age, sex, race/ethnicity, years of education, military status, and TBI history, there were no statistically significant differences in Aβ38, Aβ40, Aβ42, pTau-181, pTau-217, pTau-231, or pTDP43 between groups (*p* = 0.06–0.85).

**Table 6.** General Linear Model Comparing Participants with Repeated Traumatic Brain Injury History and Controls on Plasma Biomarkers

	TBI (*n* = 22)	Control (*n* = 8)	ηρ^2^	*p*
Aβ38	10.1 ± 0.1	9.4 ± 0.3	0.14	0.07
Aβ40	13.0 ± 0.8	12.9 ± 0.2	0.01	0.64
Aβ42	13.3 ± 0.1	13.3 ± 0.2	0.00	0.85
pTau-181	13.0 ± 0.2	12.2 ± 0.4	0.11	0.11
pTau-217	11.0 ± 0.2	10.6 ± 0.3	0.04	0.35
pTau-231	13.2 ± 0.2	12.6 ± 0.3	0.10	0.14
pTDP43	10.3 ± 0.1	10.8 ± 0.2	0.15	0.06

Final models from stepwise forward linear regression procedures to associate neuroimaging biomarkers with symptom domains can be viewed in Table 7. Amygdala normative percentile and/or amygdala asymmetry index were included in the final models for NSI, SWLS, PCL5, BIS, BPAQ, and ISI. Only two models included a statistically significant PET outcome in the final model: tau uptake in Braak regions 3 and 4 for PCL5 scores and PiB in the orbitofrontal and parietal regions for ISI scores. Models for BSI and ESS failed to converge, indicating no neuroimaging biomarkers met inclusion criteria.

**Table 7.** Included Neuroimaging Biomarkers from Stepwise Forward Linear Regression Models

	Included variables	β	*p*	Adjusted *R*^2^	*p*
NSI	*Posterior cingulate percentile*	−0.37	0.07	0.19	0.046*
	*Amygdala asymmetry index*	0.35	0.08		
SWLS	*Amygdala asymmetry index*	−0.55	0.007*	0.27	0.007*
PCL5	*Hippocampus percentile*	0.53	0.003*	0.60	<0.001*
	*Amygdala percentile*	0.44	0.009*		
	*Tau Braak 3–4*	−0.50	0.005*		
	*PiB parietal*	0.32	0.054		
	*Thalamus percentile*	−0.28	0.079		
BIS	*Amygdala percentile*	0.41	0.039*	0.25	0.023*
	*Entorhinal cortex percentile*	0.35	0.071		
BPAQ	*Amygdala percentile*	0.44	0.037*	0.15	0.037*
ISI	*PiB orbitofrontal*	0.55	0.005*	0.46	0.004*
	*PiB parietal*	−0.60	0.003*		
	*Amygdala asymmetry index*	0.58	0.006*		
	*Amygdala percentile*	−0.43	0.029*		

NSI, Neurobehavioral Symptom Inventory; ISI, Insomnia Severity Index; PCL5, PTSD Checklist for DSM-5; BSI, Brief Symptom Inventory; SWLS, Satisfaction with Life Scale; BIS, Barratt Impulsivity Scale; BPAQ, Buss Perry Aggression Questionnaire. ^*^statistically significant at *p* < 0.05.

## Discussion

Repeated TBIs are a risk factor for later life neurodegenerative disease, but the spatiotemporal development of trauma-related neurodegeneration is not clear. Natural history studies of AD show that certain biomarkers (e.g., amyloid PET, plasma Aβ42, p-Tau217, p-Tau181, or pTau231) become abnormal far prior to the development of symptoms.^5^ In this sample with a mean age of 40 and a history of 5+ TBIs, these core diagnostic biomarkers for AD were not different from controls despite significantly higher symptom burden. Moreover, the TBI group had volumes of critical brain regions larger than typical diagnostic cutoffs for AD (i.e., ∼5^th^ percentile or lower). In this sample at risk for trauma-related neurodegeneration, the clinical and biological presentation is not consistent with preclinical or prodromal AD.^38^

It is possible that the younger average age of the sample (∼40 years) is too early to detect AD-like changes or other aspects of neurodegeneration. However, early-onset AD has been diagnosed in patients within this age range and other neurodegenerative diseases, such as chronic traumatic encephalopathy, have been diagnosed in younger patients (postmortem in the case of chronic traumatic encephalopathy).^39,40^ Furthermore, abnormal Aβ is routinely detected in the fourth and fifth decade of life in patients without symptoms who go on to develop AD.^5,38^ Given how symptomatic the current cohort was, one would expect a more substantial biomarker profile suggestive of AD. Altered brain connectivity is a potential alternative explanation for the elevation in symptoms years after TBI in comparison with controls. Functional mapping of brain connectivity was not used in the current study, but prior work has suggested that alterations to certain networks following TBI are strongly associated with chronic symptoms, such as depression.^41^

### Limitations

Recruitment for this study was challenging due to COVID-19 pandemic, resulting in the possibility that analyses were underpowered. To offset the smaller sample size, effect sizes are presented for all analyses and each model underwent a bootstrap procedure to enable more robust estimation. The groups did not have equal representation across demographics, especially in the context of Veteran status. To control for these group differences, Veteran status and several other covariates were included in all models. This is a cross-sectional study, so it is not known what the participant’s baseline volumetrics or symptoms were prior to injury. It is also not known if participants in this study sought treatment following their TBIs, or how they managed the injuries.

## Conclusion

In this cross-sectional study of ∼40-year-old participants with repeated TBI history compared with controls, notable elevations were observed for those with repeated TBI history in symptoms of concussion, post-traumatic stress, psychological distress, aggression, sleepiness and insomnia 31 years following their first TBI and 8 years from the most recent TBI. However, no group differences were observed in neurocognitive performance, MRI volumetrics, plasma proteins related to neurodegenerative disease, or Aβ/tau PET scans. Several volumetrics in critical brain regions were associated with six symptom domains in the TBI group while PET scan outcomes were only associated with two symptom domains. These results may suggest that cortical volume and/or asymmetry indices (especially in the amygdala) may be a more viable early biomarker of chronic symptom burden in this population than PET scans. Longitudinal studies with repeated assessment of neurodegenerative biomarkers are necessary to understand the clinical development of these markers (in relation to clinical presentation) over time in a population with a history of TBIs.

## Data Availability

De-identified data are available by request from the Federal Interagency TBI Research Informatics System.

## Transparency, Rigor, and Reproducibility Statement

This is a secondary analysis of a cohort study so the study was not pre-registered. The analysis plan for the current study was not pre-registered online but was conceived by the primary authors. The primary author certifies that the analyses were pre-specified. The sample size was a convenience sample of participants enrolled at a single site. Data collection and analyses were performed by investigators who were aware of relevant characteristics. Data and analytic codes are available at reasonable request. R was used to complete analyses.

## Abbreviations Used

AB: amyloid beta
AD: Alzheimer's Disease
BIS: Barratt Impulsiveness Scale
BPAQ: Buss Perry Agression Questionnaire
BSI-18: Brief Symptom Inventory-18
COWAT: Controlled Oral Word Association Test
CVLT: California Verbal Learning Test
ESS: Epworth Sleepiness Severity
ISI: Insomnia Severity Index
MRI: magnetic resonance imaging
NSI: Neurobehavioral Symptom Inventory
PCL-5: PTSD Checklist for DSM-5
PET: positron emission tomography
PiB: Pittsburgh Compound B
PSI: Processing Speed Index
pTau: Phosphorylated tau
PTSD: Post Traumatic Stress Disorder
ROI: region of interest
SUV: Standardized Uptake Value
SWLS: Satisfaction with Life Scale
TBI: traumatic brain injury
TMT: Trail Making Test
WAIS: Wechsler Adult Intelligence Scale
