# Supplementary material for: Identifying a Biological Signature of Trauma-Related Neurodegeneration Following Repeated Traumatic Brain Injuries Compared with Healthy Controls
Source: Neurotrauma Rep. 2025 Jul 2;6(1):560–8. doi: 10.1089/neur.2025.0052 (PMC12235121; doi:10.1089/neur.2025.0052)
Supplement: Supplementary Table S1 [file neur.2025.0052_supp_tables1.docx]

| **Supplemental Table 1. Qualitative interpretation of positron emission tomography scans for Pittsburgh Compound B (PiB) and AV-1451 by a clinical neuroradiologist** | | | | |
| --- | --- | --- | --- | --- |
|  |  | **TBI** | **Control** |  |
| **PiB** | **Cerebellum** | Definitely Normal (17/17; 100%) | Definitely Normal (5/5; 100%) |  |
|  | **Temporal Lobe** | Definitely Normal (17/17; 100%) | Definitely Normal (5/5; 100%) |  |
|  | **Occipital Lobe** | Definitely Normal (17/17; 100%) | Definitely Normal (5/5; 100%) |  |
|  | **Frontal Lobe** | Definitely Normal (17/17; 100%) | Definitely Normal (5/5; 100%) |  |
|  | **Parietal Lobe** | Definitely Normal (17/17; 100%) | Definitely Normal (5/5; 100%) |  |
|  | **Basal Ganglia** | Definitely Normal (17/17; 100%) | Definitely Normal (5/5; 100%) |  |
|  | **Cingulate** | Definitely Normal (17/17; 100%) | Definitely Normal (5/5; 100%) |  |
| **AV1451** | **Cerebellum** | Definitely Normal (17/17; 100%) | Definitely Normal (5/5; 100%)  Probably Normal (1/5; 100%)  Definitely Abnormal (1/5; 100%) |  |
|  | **Temporal Lobe** | Definitely Normal (13/17; 76.5%)  Probably Normal (4/17; 23.5%) | Definitely Normal (3/5; 60%)  Probably Normal (1/5; 5.9%)  Probably Abnormal (1/5; 5.9%) |  |
|  | **Occipital Lobe** | Definitely Normal (17/17; 100%) | Definitely Normal (5/5; 100%) |  |
|  | **Frontal Lobe** | Definitely Normal (15/17; 88.2%)  Probably Normal (1/17; 5.9%)  Probably Abnormal (1/17; 5.9%) | Definitely Normal (4/5; 80%)  Probably Abnormal (1/5; 20%) |  |
|  | **Parietal Lobe** | Definitely Normal (17/17; 100%) | Definitely Normal (5/5; 100%) |  |
|  | **Basal Ganglia** | Definitely Normal (17/17; 100%) | Definitely Normal (5/5; 100%) |  |
|  | **Cingulate** | Definitely Normal (17/17; 100%) | Definitely Normal (5/5; 100%) |  |
